# Menopause-Associated Lipid Metabolic Disorders and Foods Beneficial for Postmenopausal Women

**DOI:** 10.3390/nu12010202

**Published:** 2020-01-13

**Authors:** Seong-Hee Ko, Hyun-Sook Kim

**Affiliations:** Department of Food and Nutrition, College of Human Ecology, Sookmyung Women’s University, Seoul 04310, Korea; marialovegod625@gmail.com

**Keywords:** menopause, estrogen deficiency, lipid metabolic disorder, beneficial foods

## Abstract

Menopause is clinically diagnosed as a condition when a woman has not menstruated for one year. During the menopausal transition period, there is an emergence of various lipid metabolic disorders due to hormonal changes, such as decreased levels of estrogens and increased levels of circulating androgens; these may lead to the development of metabolic syndromes including cardiovascular diseases and type 2 diabetes. Dysregulation of lipid metabolism affects the body fat mass, fat-free mass, fatty acid metabolism, and various aspects of energy metabolism, such as basal metabolic ratio, adiposity, and obesity. Moreover, menopause is also associated with alterations in the levels of various lipids circulating in the blood, such as lipoproteins, apolipoproteins, low-density lipoproteins (LDLs), high-density lipoproteins (HDL) and triacylglycerol (TG). Alterations in lipid metabolism and excessive adipose tissue play a key role in the synthesis of excess fatty acids, adipocytokines, proinflammatory cytokines, and reactive oxygen species, which cause lipid peroxidation and result in the development of insulin resistance, abdominal adiposity, and dyslipidemia. This review discusses dietary recommendations and beneficial compounds, such as vitamin D, omega-3 fatty acids, antioxidants, phytochemicals—and their food sources—to aid the management of abnormal lipid metabolism in postmenopausal women.

## 1. Introduction

### 1.1. What Is Menopause?

Clinically, menopause is diagnosed when a woman has not menstruated for one year due to the loss of ovarian follicular activity, which typically occurs at around 45–55 years of age [1]. In the USA, spontaneous menopause occurs at 51 years of age, on an average. Generally, women live longer than men, and the global average life span of women has been increasing. The median age of women has been increasing gradually and is expected to reach 82 years by 2025 in developed countries. Therefore, approximately one-third of women’s lives would be lived after menopause [2]. Menopause occurs over several years and not at a single point in time. It is preceded by a stage where women experience irregular menstrual cycle, referred to as menopausal transition (perimenopause), which involves coping with the cessation of oocyte production in the ovaries [3].

Hormonal change is among the major physiological changes associated with menopause. Estrogen, a primary female sex hormone, dictates the secondary sex characteristics and affects the development and functioning of the female reproductive system. All estrogens are C18 steroids [4]. In addition to its indispensable role in the development of female reproductive tissues and organs—such as breasts, vagina, and uterus—estrogen is also involved in maintaining the function of these tissues and organs during puberty, adulthood, and pregnancy. Estradiol (E2) is almost always present in the body of women of reproductive age, whereas estriol (E3)—which is produced by the placenta—is primarily abundant during pregnancy and plays an important role in maintaining early-stage pregnancy [4]. During pregnancy, the E3 levels increase up to 1000-fold, while the estrone and E2 levels increase up to 100-fold. Thus, E3 can be measured in urine. Throughout the follicular phase of the menstrual cycle, the thecal cells produce androstenedione, which functions as a metabolic precursor to estrone and testosterone in the ovaries and peripheral tissues [5].

### 1.2. Dysregulation of Lipid Metabolism Due to Alterations in E2

During a woman’s fertile life, the average level of total estrogen is 100–250 pg/mL. However, the concentration of E2 in circulation declines up to 10 pg/mL postmenopause [6]. This hormonal menopause is associated with pathological menopausal syndromes, such as disturbances in sleep/mood, vasomotor symptoms (including hot flashes and night sweats), urogenital atrophy, osteopenia and osteoporosis, psychiatric disorders, sexual dysfunction, skin lesions, cardiovascular diseases (CVDs), cancer, metabolic disorders, and obesity [7]. Women are at a higher risk of developing CVDs after menopause due to estrogen deficiency and dysregulated lipid metabolism [8]. Estrogens, especially E2, exert a protective role in the cardiovascular system and are produced primarily in the ovaries via a process that uses low-density lipoprotein (LDL) cholesterol (LDL-C) as a substrate. However, circulatory LDL-C cannot be utilized to synthesize estrogen during menopause, thereby resulting in decreased estrogen production. Therefore, menopause is associated with increased blood LDL-C levels and enhanced CVD risk [9].

Experimental data have demonstrated that E2 plays an essential role in the β-oxidation of fatty acids in the mitochondria. In an aromatase (estrogen synthase) knockout mouse model, E2 was not detectable in the liver tissue; additionally, decreased expression of mRNA was observed, along with a lowered activity of enzymes involved in fatty acid metabolism. Importantly, E2 treatment resulted in recovered mRNA expression and increased the activity of enzymes involved in fatty acid metabolism in an estrogen synthase knockout mouse model [10]. In a postmenopausal model, female mice (aged 2–4 weeks)—after ovariectomy—exhibited decreased expression of nuclear receptors and proteins needed for efficient energy expenditure, such as peroxisome proliferator-activated receptor (PPAR) gamma γ, PPARδ, PCG1α, PCG1β, and ERR1—in the adipose tissue and muscle—relative to that in the sham-operated control mice. Additionally, the ovariectomized (OVX) mice exhibited decreased expression of enzymes involved in the β-oxidation of fatty acids and transcription factors required for lipolysis [11]. Therefore, the depletion of E2 by ovariectomy or after menopause may downregulate the expression of genes required for efficient energy expenditure as fuel sources in the human body and genes involved in fatty acid metabolism or lipid catabolism, which may induce obesity or metabolic disorders in postmenopausal women [12].

Menopause-induced estrogen deficiency may also lead to various metabolic disorders, including dysregulated lipid metabolism. This review has two distinctive parts. The first part of the review comprehensively summarizes the definition of menopause and postmenopausal physiology, including hormonal change and dysregulation of lipid metabolism caused primarily due to alterations in E2 levels. The second half of the review provides practical and applicable daily dietary recommendations and proposes food sources that would prove to be beneficial for postmenopausal women.

## 2. Lipid Metabolic Disorders Associated with Menopause

### 2.1. Menopause-Associated Changes in Fat Mass and Fatty Acid Metabolism

Aging is associated with changes in body composition, which affect physical activity and health in humans [13]. Most studies suggest that the changes in the composition of the female body coincide with menopause. Some of these changes can be partly explained by the impairment of the protective role of estrogens and a relative increase in circulating androgen levels [14]. Previous studies have demonstrated that endogenous sex hormones can influence the lipid profile in premenopausal and postmenopausal women because the receptors for estrogen and androgen are expressed in both visceral and subcutaneous adipocytes. Therefore, changes in the levels of endogenous sex hormones may affect lipid metabolism in the fat tissues of middle-aged women [15]. Postmenopausal women are also reported to exhibit higher total body fat mass, fat percentage, and accumulation of central fat than premenopausal women [16]. Kim et al. reported that although both premenopausal and postmenopausal women exhibited similar mean body mass index (BMI) values, postmenopausal women exhibited larger waist circumference [17]. A controlled longitudinal study reported that postmenopausal women were at a significantly higher (2.88-fold) risk of developing abdominal obesity than premenopausal women [18,19].

During menopausal transition—that usually spans 2–7 years—clinical changes occur in the body composition due to aging and hormonal changes [20]. Ovarian estrogens increase the storage of peripheral fat mainly in the gluteal and femoral subcutaneous regions, while androgens—primarily bioavailable testosterones—augment the accumulation of visceral abdominal fat. The marked decrease in estrogen concentrations accompanying relative hyperandrogenism is regarded as the main factor that causes weight gain and redistribution of body fat in postmenopausal women [21].

A longitudinal community-based Study of Women’s Health Across the Nation (SWAN)—a five-year follow-up study—reported that relative androgen excess (a higher baseline testosterone/E2 ratio) can predict incident of metabolic syndrome, including dysregulated lipid metabolism and obesity, during menopausal transition [22]. This study reported that postmenopausal women had 2-fold higher visceral abdominal fat and subcutaneous adipose tissue (SAT) than premenopausal women. However, the testosterone levels were similar among premenopausal and postmenopausal women. This suggested that fat redistribution may be affected by a marked decreased in estrogen levels but not by increased testosterone level [23]. Estrogen and androgen receptors are expressed in visceral and subcutaneous fat cells. Therefore, changes in the levels of sex hormones can influence lipid metabolism [24]. However, the levels of sex hormone-binding globulin also decrease at the onset of menopause, which increases the levels of bioavailable testosterone. Thus, several studies have suggested that augmented bioavailable testosterone can directly regulate the accumulation of visceral fat through androgen receptors in the adipocytes of the abdominal fat [25]. Additionally, Ziaei et al. reported that free testosterone correlated with the BMI and waist circumference in postmenopausal women [26]. Oral administration of androgens to postmenopausal women preferentially augmented the levels of visceral abdominal fat [27].

Excessive visceral abdominal fat can cause metabolic alterations, especially in fatty acid metabolism, in postmenopausal women. Visceral fat is reported to be associated with a high lipolysis rate (breakdown of triacylglycerol (TG) into glycerol and free fatty acid (FFA)), which results in an increased flux of FFA to the liver and enhanced hepatic insulin resistance. One of the strategies for treating insulin resistance involves elimination of the excess FFAs [21,28]. The adipose tissues of postmenopausal and premenopausal women exhibit differential lipid metabolism. The basal lipolysis rate in the gluteal adipose tissue of postmenopausal women was 77% lower than that in the gluteal adipose tissue of premenopausal women. Further, the activity of adipose tissue lipoprotein lipase (AT-LPL)—which catalyzes the conversion of TG into FFAs for uptake and storage of TG by the adipocytes and plays a major role in the accumulation and distribution of fat stores—was significantly higher in the gluteal and abdominal adipose tissues of postmenopausal women in than that of premenopausal women. Thus, lower lipolysis rate and higher AT-LPL activity may predispose postmenopausal women to increased accumulation of body fat after menopause [29,30]. Takahashi et al. comparatively evaluated fatty acid metabolism between premenopausal and postmenopausal women. Interestingly, postmenopausal women exhibited significantly higher concentrations of fatty acid metabolites, such as heptanoate (C7:0), octanoate (C8:0), and pelargonate (C9:0), in the visceral fat (but not in subcutaneous fat) than premenopausal women. This may result in enhanced fatty acid metabolite accumulation in the visceral fat due to elevated lipolysis. The expression of transcripts encoding adiponectin, PPAR-γ, and fatty acid transporter in the gluteal fat of estrogen-depleted postmenopausal women was significantly higher than in the gluteal fat of premenopausal women. These findings indicate that gluteal fat may be insulin-resistant and might contribute to the accumulation of body fat after menopause, which may be related to the development of metabolic syndrome [30,31]. Metabolic syndrome is defined as a cluster of conditions characterized by impaired glucose metabolism, high blood pressure, central obesity, and low LDL-C and high triglyceride levels [32]. Recent studies indicate that metabolic syndrome is more prevalent among postmenopausal women than premenopausal women [33]. Importantly, half of the postmenopausal, Iranian women have been reported to suffer from metabolic syndrome [34]. Therefore, preventive strategies must be devised for combating the impaired metabolic changes in postmenopausal women.

### 2.2. Menopause-Associated Changes in Fat-Free Mass (FFM) and Basal Metabolism

One of the noticeable changes in body composition that is associated with the age of women is the loss of FFM, or lean body mass (LBM). Generally, there is increased intra-abdominal adipose tissue and decreased fat in the hip–thigh area among women [31]. Many cross-sectional studies using dual-energy X-ray absorptiometry (DEXA) have reported that postmenopausal women exhibit lower FFM or LBM in the whole body, trunk, and lower extremity regions than premenopausal women [35]. The loss of FFM or LBM is likely to coincide with menopausal transition, which suggests that sarcopenia may be related to the menopausal status [36]. Sarcopenia refers to the degenerative loss of skeletal muscle that occurs at a rate of 3%–8% every 10 years after the age of 30 years, and accelerates with age. This condition is associated with increased risk of functional disability, falls, fracture, and overall mortality among the elderly [37]. Women develop sarcopenia earlier than men, and the incidence of sarcopenia increases rapidly during the menopausal period [38]. Interestingly, a six-year follow-up study by Poehlman et al. reported that menopausal women lost more FFM than age-matched premenopausal women [36]. Recently, Kim et al. reported that one out of five relatively healthy Korean menopausal women aged over 65 years exhibited a decrease in muscle mass, and 7.6% of the women exhibited a decrease in muscle mass and strength. The study also reported that sarcopenia also intensified with aging [39].

The loss of FFM or skeletal muscle induces an age-related decline in the basal metabolic rate (BMR) [40]. The loss of FFM or LBM and gain of adipose tissue can alter the BMR. The BMR or resting energy expenditure (REE)—which is the product of energy exchanges occurring in all human cells—is the amount of energy—in calories—required to maintain biological life functions, including the regulation of body temperature, contraction and relaxation of muscles, breathing, blood circulation, cell growth, and functions of the brain and neurons [16]. Generally, BMR constitutes approximately 60%–75% of the diurnal calorie expenditure. However, BMR can vary highly among individuals [41]. FFM or skeletal muscle accounts for approximately 60%–85% of the body mass, which is regarded as an energy consumer and a major determinant of REE [42], whereas fat mass acts as a storage site for surplus energy. Therefore, changes in the body composition in women—around the menopausal period—such as the loss of FFM leads to a decrease in the BMR. Thus, the physical body of menopausal women expends less energy to maintain the basic life processes [43].

Although the causality between the incidence of sarcopenia and menopausal status is still controversial [44], it is generally accepted that natural age-related menopause is associated with an accelerated loss of FFM or skeletal muscle, which is associated with decreased energy expenditure during rest and physical activity [45]. These changes may indicate alterations in energy metabolism.

## 3. Menopause-Associated Changes in Lipid Metabolism

### 3.1. Lipids: Lipoproteins, Apolipoproteins, LDL-C, High-Density Lipoprotein (HDL) Cholesterol (HDL-C), and TGs (Triglycerides)

#### 3.1.1. Lipoproteins

Lipoproteins, which comprise proteins and lipids, play a major role in transporting the endogenous lipids. These are circulating lipids that are not directly absorbed from the intestine but are processed through various tissues, especially the liver. The lipoprotein fractions are classified as follows, based on increasing density: chylomicrons, very-low-density lipoproteins (VLDLs), LDLs, and HDLs7 [46,47].

#### 3.1.2. Apolipoproteins

Apolipoproteins are the protein components of lipoproteins that stabilize the lipoproteins when they pass through the blood. Additionally, apolipoproteins are involved in several important functions, such as conferring specificity to the lipoprotein complexes, which are recognized by specific receptors expressed on cell surfaces. 

#### 3.1.3. LDL-C

LDL fraction—a major carrier of cholesterol—binds about 60% of the total serum cholesterol. The function of LDL is to transport cholesterol to the tissues. Additionally, LDL-C forms a part of the plasma membrane or can be converted into various metabolites, including steroid hormones [28]. Therefore, the function of LDL is not restricted to depositing cholesterol and other lipids in the peripheral cells. Furthermore, peripheral cells also express the LDL receptor, apoB-100. As LDL-targeted cells include cells of the vascular endothelium, high concentration and activity of LDL have serious implications in the etiology of CVDs [48]. LDL contributes to the formation of plaque, which is a thick, hard deposit that can clog the arteries, thereby compromising the flexibility of arteries, resulting in atherosclerosis [49]. Patients with type 2 diabetes exhibit dyslipidemia characterized by low HDL and high TG levels due to insulin resistance and increased levels of LDL [50].

#### 3.1.4. HDL-C

The HDL fraction of serum lipoproteins inhibits the deposition of LDL-C. The major functions of HDL are removing unesterified cholesterol (free cholesterol) from cells and removing cholesterol from other lipoproteins. HDL delivers the cholesterol accumulated in various cells and lipoproteins to the liver, where it is excreted via bile [51]. This is accomplished via two key properties of HDL. The first is that HDL can bind to the receptors expressed on both hepatic and extrahepatic cells. These cells contain receptors specific for HDL or LDL that can be bound by HDL through its apoE component. The second property of HDL is by virtue of its apoA-1 component, which activates LCAT (lecithin-cholesterol acyltransferase, also called phosphatidylcholine–sterol O-acyltransferase) [51], an enzyme that converts free cholesterol into cholesteryl esters. The free cholesterol (recipient) substrate is derived from the plasma membrane of cells or surfaces of other lipoproteins. Subsequently, the cholesteryl esters can be exchanged between the plasma lipoproteins, which is mediated by a transfer protein, cholesterol ester transfer protein (CETP). LCAT-mediated conversion of free cholesterol to cholesteryl esters facilitates the delivery of cholesterol from nonhepatic cells as well as from other lipoproteins [51]. The cholesteryl esters can be directly transported to the liver by HDL, or indirectly by LDL after the ester is transferred from HDL to LDL by CETP. The esters accumulated in the liver cells can be hydrolyzed by cholesterol esterase to release free cholesterol, which is excreted as a bile salt in the bile through a process called reverse cholesterol transport. This is the main pathway for cholesterol excretion from the body [52]. HDL decreases the levels of accumulated cholesterol in the endothelium of blood vessels, which is beneficial for the cardiovascular system and aids in preventing the formation of fatty plaques and atherosclerosis [52]. One-fourth to one-third of blood cholesterol can be transported by HDL. Thus, an optimal HDL level may protect against heart attack and stroke, while low HDL levels are reported to increase the incidence of coronary heart disease (CHD) [53].

#### 3.1.5. Triglycerides

TGs (Triglycerides) are another type of fats that differ from lipoproteins in that TGs are involved in energy metabolism. Most stored body fat is in the form of TGs, which represent a highly concentrated form of energy and account for nearly 95% of dietary fat. To generate energy, fatty acids are released in nonesterified form, which is the FFAs, from TGs in the adipose tissue. The FFAs are then transported by binding albumin to various tissues for oxidation. Elevated blood TG levels are reported to be associated with CVDs, type 2 diabetes, and atherosclerosis [54].

### 3.2. Menopause-Associated Alterations in Various Lipids

Menopausal women are at high risk for developing CVDs due to dysregulated lipid metabolism and estrogen deficiency [55]. The SWAN study involved 2659 women with baseline ages ranging from 42 to 52 years who were in the premenopausal or menopausal transition states. The study reported that the alterations in various body fats were related to the changes in the menopausal status and concentrations of E2 and follicle-stimulating hormone (FSH). The alterations in various fats were initiated during the late phases of menopause. The levels of total cholesterol, LDL, triglycerides, and lipoprotein peaked during menopausal transition and the early postmenopausal stage [22,25]. The SWAN study also reported that the middle-aged women with the highest quartile E2 levels had the lowest levels of total cholesterol and LDL, whereas women with the lowest quartile FSH levels had the highest levels of total cholesterol and LDL-C. Lower levels of E2 and sex hormone-binding globulin were associated with medium to low LDL concentrations, while higher levels of FAI (free androgen index) were associated with higher cholesterol, TG, and LDL levels and lower HDL levels in middle-aged premenopausal women [56]. The changes in sex hormone levels and lipid metabolism may also increase the risk of CHD in women [23].

Moreover, several studies have reported that there is a positive correlation between menopausal status and high levels of total cholesterol, LDL, apo-B, and high total cholesterol-to-HDL ratio [57,58]. Recently, Anagnostis et al. reported that the total cholesterol-to-HDL ratio also increased with menopause. The total cholesterol-to-HDL ratio is a better indicator of CVD than total cholesterol itself [59]. A community-based cohort study explored the changes in serum lipid profile during menopausal transition in Chinese women. In total, 593 healthy women aged 35–64 years were followed-up annually for up to 3 years. Menopausal transition in these women exhibited a correlation with the serum lipid profile. The total cholesterol, TG levels, and total cholesterol-to-HDL ratio peaked in women exhibiting menopausal transition compared with those in the premenopausal women. Contrastingly, there was no significant change in HDL-C among the different menopausal status groups [60]. LDL particle size and density also changed with the menopausal status. The levels of small and dense LDL particles increased from 10%–13% in premenopausal women to 30%–49% in postmenopausal women [16]. The small and dense LDL is reported to be an important determinant of atherosclerosis and CVDs in postmenopausal women [61].

### 3.3. Correlation between Menopausal Status and HDL-C

Some studies have reported that HDL-C levels increase after menopause. The longitudinal SWAN study reported that the HDL-C levels increase gradually from premenopause and peak during menopausal transition. Subsequently, HDL-C levels gradually decline till late postmenopause [57]. However, some studies have indicated a decrease in the levels of plasma HDL-C. A cohort study on 541 healthy middle-aged women followed up through menopause reported that postmenopausal women exhibit a decline in HDL-C levels (mean, 0.53–0.43 mmol/L) and a gradual increase in LDL-C levels (mean, 3.14–3.33 mmol/L) [62]. Similarly, the Women’s Health in the Lund Area (WHILA) study involving 6908 women reported that postmenopausal women exhibit significantly lower levels of plasma HDL-C than premenopausal women [63]. Additionally, follow-up data from the Healthy Women Study demonstrated that menopausal transition was associated with decreased HDL-C levels and increased LDL-C and TG levels relative to that in the postmenopausal stage [64]. The changes in HDL-C accompanying the menopausal status may be more complex than that indicated by the measurement of plasma levels of total HDL-C. Recent studies have suggested that the changes in the proportion of HDL subclasses are more strongly associated with the menopausal status than the changes in the total HDL-C. Most of the available evidence suggests that menopausal transition period and menopause are characterized by increased levels of small HDL subfraction 3 cholesterol (HDL 3-C) and decreased levels (by 25%) of large, buoyant, cardioprotective HDL subfraction 2 cholesterol (HDL 2-C). Additionally, the levels of small dense LDL particles increased up to 30%–49% in menopause [65]. Some studies suggest that HDL 2-C is more active in reverse cholesterol transport and has better antiatherogenic properties than HDL 3-C [52].

### 3.4. Adiposity Associated with Menopause

The degree of accumulation of body fat is referred to as adiposity, which is commonly used to indicate excess body fat [66]. A well-documented metabolic phenotypic feature observed after menopause is an increased tendency for body fat deposition in the abdominal region with greater waist circumference [43]. The rapid decline in ovarian function and the subsequent decline in the production of circulating steroid-based sex hormones—such as estrogen—associated with menopause results in increased overall adiposity, especially the abdominal visceral adiposity [67,68]. Increased adiposity in postmenopausal women is significantly associated with hyperinsulinemia, which suggests that insulin resistance may be responsible for the development of the key features of postmenopausal dyslipidemia [69], obesity [70], metabolic syndrome [71], and type 2 diabetes [72].

Age-related loss of ovarian hormones was examined in human (menopause) and animal models. Menopause induces the loss of ovarian hormones that contributes to alterations in the distribution of adipose tissue. The significant decrease in both estrogen and progesterone due to follicular depletion results in a more androgenic pattern of fat distribution (central or abdominal adiposity) [20]. OVX mice—a surgical menopause model—exhibit a significant increase in body weight due to reduced energy expenditure and increased food consumption. Additionally, several clinical disorders, such as increased peripheral inflammation, augmented insulin resistance, and magnified adipose tissue cells, have also been observed in the OVX mice [73,74]. However, the OVX mice recovered from these clinical features upon being supplemented with estrogen (E2). Estrogen is also reported to exhibit a strong inhibitory effect on key adipogenic genes, such as PPAR-γ, CEBPb (CCAAT-enhancer-binding proteins), and lipin1 in non-OVX female and OVX female mice supplemented with estrogen [74]. 

The function of estrogens can be transduced by nuclear receptors—which belong to the superfamily of nuclear receptors—that also act as transcription factors. There are two types of estrogen receptors: estrogen alpha (ER α) and estrogen beta (ER β) receptors [75]. Both ER α and ER β are expressed in the human subcutaneous and visceral adipose tissues [76]. The specificity of ER α lies in its ability to regulate the activity of adipose tissue cells and fat distribution underlying sexual dimorphism. Studies on male and female ER α whole-body knockout mice reported that the absence of ER α causes a near doubling of adipocyte hyperplasia and hypertrophy, insulin resistance, hyperlipidemia with smaller LDL particles, and glucose intolerance in both sexes, which suggests that estrogens play a protective role against these phenomenon [77,78]. The knockdown of adipocyte-specific ER α (Adipo ER α) is reported to increase the body weight, adipose tissue mass, and adipocyte size in females. This indicated that adipocyte ER α regulates body fat distribution, adipose tissue inflammation, and fibrosis. Thus, adipocyte ER α is necessary for mediating the beneficial effects of estrogen on metabolism [79].

Adipokines such as leptin, adiponectin, resistin, and ghrelin can modulate the size and number of adipocytes and angiogenesis through the paracrine system, and are thus involved in regulating the fat mass [80]. Moreover, adipokines are also involved in the regulation of blood pressure, blood clot formation, lipoprotein metabolism, immune function, and inflammatory reactions [81]. Menopause is associated with marked elevation in the levels of leptin and resistin and downregulation of ghrelin and adiponectin. Postmenopausal women with a history of hypertension exhibited increased blood leptin levels [82]. Further, postmenopausal women with low bone mineral density exhibited significantly high circulatory levels of adipocyte-secreted hormones (adiponectin, resistin, PAI-1, and adipsin) [83]. This indicated that altered adipokine levels may play a crucial role in the pathogenesis of metabolic disorders, such as hypertension, CVD, and osteoporosis. High leptin levels along with low adiponectin levels exert a positive effect on insulin resistance markers. Thus, adipocytokine levels may account for postmenopausal dyslipidemia resulting from insulin resistance [84].

### 3.5. Obesity Associated with Menopause

Obesity is mostly defined as per the established classification metrics with respect to the BMI [66,85] and as an abnormal or excess total body fat that presents a health risk [86]. BMI is a simple index of weight-to-height (kg/m^2^), and the World Health Organization (WHO) defines obesity as a BMI ≥ 30 kg/m^2^, overweight as BMI 25–29.9 kg/m^2^, and underweight as BMI < 18.5 kg/m^2^ [86]. Obesity is a precursor condition for various disorders, such as CVD, metabolic syndrome, and type 2 diabetes [87,88]. It is caused by a combination of both hereditary and environmental factors [89]. Women in midlife exhibit continuous weight gain. However, the reasons for increased obesity among menopausal women are not fully understood and are contradictory. There are several inconsistencies among studies that have reported a correlation between body mass and age among postmenopausal women due to differences in study design, analysis, or the verifying control of confounding variables [12]. 

One of the major reasons for increased obesity among menopausal women is the decreased level of estrogens. Obesity is associated with a higher probability of surgical menopause, where the ovaries are surgically removed by bilateral oophorectomy [2]. Clegg et al. demonstrated that the absence of estrogens may be an important obesity-triggering factor [90]. Moreover, some studies have reported that estrogen deficiency enhances metabolic dysfunction and predisposes the individual to type 2 diabetes, metabolic syndrome, and CVD [16]. Aromatase (estrogen synthase) knockout mice—that cannot synthesize E2—are obese and insulin-resistant [91]. As described previously, ERα appears to be a key regulator of obesity because ERα knockout mice exhibit increased obesity and glucose intolerance compared with wild-type female mice [77,92]. A recent study reported that postmenopausal women who have received aromatase inhibitors for the treatment of breast cancer exhibited higher body fat percentage and insulin resistance than control subjects without a history of breast cancer [93].

A reduction in energy expenditure during midlife can also cause obesity during menopause. According to a four-year follow-up study, women undergoing menopause exhibited a greater decrease in energy expenditure than premenopausal women [94], which may be attributed to the reduction in physical activity. The loss of LBM leads to a reduction in basal energy metabolism with aging [95]. A population-based cross-sectional study involving 292 Brazilian women reported that a higher risk of overweight/obesity was associated with (odds ratio (OR), 2.1; 95% confidence interval (CI) 1.233–3.622; *p* = 0.006) a waist circumference >88 cm (OR, 1.7; 95% CI 1.054–2.942; *p* = 0.03) for inactive women. Even after adjustment for age, menopause status, smoking, and hormone therapy, inactive women were associated with a higher risk of diabetes mellitus (OR, 2.7; 95% CI 1.233–6.295; *p* = 0.014) and metabolic syndrome (OR, 2.5; 95% CI 1.443–4.294; *p* = 0.001). These results suggest that the reduction in energy expenditure due to a sedentary lifestyle before menopause may be associated with an increased risk of developing obesity [96].

## 4. Menopause-Associated Alleviation of Dysregulated Lipid Metabolism

Dysregulation of lipid metabolism and accumulation of visceral adipose tissue after menopause can be caused by estrogen deficiency. However, the effect of diet on obesity and metabolic syndrome is still unclear. In this section, we would discuss diets that would help postmenopausal obese women introduce important nutrients and beneficial compounds in their food.

### 4.1. Dietary Recommendations

Hormonal and metabolic changes in women undergoing menopause are associated with increased body weight and visceral fat accumulation, which results in the prevalence of high abdominal obesity. As mentioned previously, insulin, total cholesterol, LDL-C, HDL-C, and total cholesterol-to-HDL ratio are usually altered in postmenopausal women with a 50% risk of having metabolic syndrome [33]. In this section, we will discuss the recommended diets for obese postmenopausal women. According to the North American reference standards for nutrients, macronutrient portions should consist of approximately 10%–35% protein-derived energy, 45%–65% carbohydrate-derived energy, and 20%–35% fat-derived energy [97]. The requirements of several key nutrients, such as protein, vitamin D, vitamin B-12, fiber, and fluid, must be met in a hypocaloric diet for older obese adults [98]. The 2013 American Heart Association (AHA)/American College of Cardiology (ACC) Guideline for Obesity prescribed the following methods to reduce food and calorie intake for weight loss: (a) 1200–1500 kcal/day for women (kilocalorie levels are usually adjusted for the individual’s body weight), (b) a 500 kcal/day or 750 kcal/day energy deficit, (c) one of the evidence-based diets that restricts certain food types (such as high-carbohydrate foods, low-fiber foods, or high-fat foods) to create an energy deficit and reduce food intake. One of the above methods can be used to reduce food and calorie intake [99].

The current recommended dietary allowance (RDA) for dietary protein intake is 0.8 g/kg/day for adults [100]. As per dietary reference intakes for Koreans 2015, the current dietary recommendation for protein is 40–45 g/day for Korean women [101]. However, the prevalence of sarcopenia was recently observed among postmenopausal women. Women develop sarcopenia earlier than men, and the incidence of sarcopenia increases rapidly during the menopausal period [102]. Therefore, the loss of skeletal muscle can lead to significant changes in amino acid metabolism as the skeletal muscle contains 53%–75% of all proteins present in the human body. Thus, low dietary protein intake may limit weight loss and result in sarcopenia. However, this can be acutely reversed using nutritional adjustments and exercise [103]. Moreover, several intervention trials have reported that the partitioning of weight loss between fat and lean mass with a greater fat-to-lean mass ratio is present in middle-aged and older women on a high-protein hypocaloric diet. Therefore, higher protein intake (1.0–1.2 g/kg/day) may compensate for some loss of muscle mass during weight loss [104,105,106].

Low-energy diet is also recommended for postmenopausal women to prevent metabolic alterations. A cross-sectional study involving 4984 Korean women reported that the traditional healthy diet involving high consumption of sea fish, seaweeds, dairy products, cereals, fresh vegetables, and fruits and low consumption of fast foods, animal fat-rich foods, sweets, and fried foods has a protective effect against the dysregulation of lipid metabolism (lower TG and higher HDL-C). A healthy diet exhibits a significant protective effect against metabolic syndrome among postmenopausal women relative to that of a Western diet, which is unbalanced and has high energy, and abundant carbohydrates and saturated fats (OR (95% CI) for highest vs. lowest quartile = 0.60 (0.41–0.86); *p* for trend = 0.004). Additionally, a healthy diet exerts protective effects against blood pressure and TG levels—during menopausal transition—and obesity and HDL-C among postmenopausal women [107].

### 4.2. Beneficial Foods and Their Sources for Postmenopausal Women

#### 4.2.1. Vitamin D

Vitamin D is a unique nutrient as it can be synthesized by the body with the help of sunlight. Therefore, vitamin D is not an essential nutrient. Most people do not require dietary vitamin D if they are exposed to sufficient sunlight every day. Vitamin D—synthesized from sunlight or obtained from food—is activated via chemical transformations in the liver and kidneys [108]. Although vitamin D can be easily obtained, recent surveys indicate that many people, particularly African Americans and Mexican Americans, suffer from vitamin D insufficiency [109]. According to a Korean National Health and Nutrition Examination Survey 2010–2011, 65.9% of Korean men and 77.7% of Korean women (age ≥ 10 years) exhibited below optimal levels of blood serum vitamin D (25-hydroxyvitamin D; 25(OH)D; 20 ng/mL) [110]. The prevalence of 25(OH)D deficiency (<10 ng/mL) was significantly higher among women than that among men [111].

It is important to understand the role of vitamin D to examine the deficiency of vitamin D. Vitamin D is the best-known nutrient among the various micronutrients and hormones which interact to regulate the balance between blood calcium and phosphorus levels. Therefore, vitamin D is essential for maintaining bone integrity [112]. Calcium is indispensable for the proper functioning of cells in all body tissues, including muscles, nerves, and glands, which obtain calcium from the blood. Vitamin D acts at three sites in the body to replenish the blood calcium levels [113]. The skeleton serves as a vast warehouse of stored calcium that can be utilized when the blood calcium levels decrease. Only two other organs can increase blood calcium, the digestive tract, which provides dietary calcium and kidneys, which recycle calcium that would otherwise be excreted in the urine. Vitamin D functions as a hormone as it is synthesized by one organ and acts on others [114]. In addition to regulation of the bone mass, vitamin D functions at the genetic level to regulate cell growth, multiplication, and specialization. Moreover, vitamin D affects over 30 body tissues, including hair follicles, reproductive system cells, and immune cells [115]. 

Studies on the role of vitamin D suggest that vitamin D deficiency may cause several diseases, including high blood pressure, CVD [116], some common cancers [117], infections (such as tuberculosis and flu) [118], inflammatory conditions, autoimmune diseases (such as type 1 diabetes and rheumatoid arthritis) [119], psoriasis [120], and multiple sclerosis [121], and that it is even associated with a higher risk of mortality [122].

A recent study reported the correlation between serum 25(OH)D levels and the alteration of lipid profile among postmenopausal women [123]. Vitamin D is a fat-soluble vitamin that can be stored in the SATs. Interestingly, Holick et al. reported that the bioavailability of vitamin D decreases in obese individuals. Thus, obesity—a major outcome of lipid metabolism dysregulation—is one of the causes of vitamin D deficiency in obesity-related diseases [109]. A cross-sectional analysis of 292 postmenopausal women aged 50–79 years in the Women’s Health Initiative Calcium–Vitamin D trial revealed that higher serum 25(OH)D levels were inversely correlated with adiposity, BMI, and waist–hip ratio. This indicated that postmenopausal obese women are susceptible to vitamin D deficiency [124]. Vitamin D deficiency is also associated with high TG levels in the blood. A cross-sectional study involving 778 postmenopausal Korean women reported that women in the lowest quartile 25(OH)D level (4.2–9.7 ng/mL) group were associated with a significantly increased risk of metabolic syndrome (OR = 2.44, 95% CI: 1.32–4.48, *p* < 0.05) compared with that in women in the highest quartile 25(OH)D level (19.9–55.9 *n*g/mL) group. Low serum 25(OH)D levels were also associated with some metabolic disorders, especially elevated blood pressure (95% CI, 1.15–2.85) and high TG level (95% CI, 1.64–4.57) [125]. The Korean National Health and Nutrition Examination Survey (KNHANES 2008–2010) revealed that postmenopausal women in the highest quartile serum 25(OH)D level group exhibited a significant decrease in the prevalence of elevated blood pressure, elevated TGs, and reduced HDL-D when compared with the postmenopausal women in the lowest quartile serum 25(OH)D level (*p* = 0.02, 0.014, and 0.002, respectively) group. This suggests that serum levels of 25(OH)D are significantly associated with a decrease in elevated blood pressure, elevated TG, and reduced HDL-C levels in postmenopausal women [126]. 

In adults, inadequate vitamin D intake may lead to poor mineralization of the bone, which results in osteomalacia, a painful bone disease in which the bones become increasingly soft, flexible, brittle, and deformed. Low vitamin D levels among elderly individuals may result in painful joints and muscles. Vitamin D deficiency also causes loss of calcium from the bones, which can result in osteoporotic fractures [127]. Vitamin D supplementation is indispensable for menopausal women as it promotes bone metabolism and prevents osteomalacia and osteoporosis [123].

A randomized controlled trial involving 76 healthy postmenopausal women with vitamin D deficiency (defined as a 25(OH)D level < 75 nmol/L)—in which the vitamin D deficiency group randomly received vitamin D 2000 IU once daily for 12 weeks—revealed that the lipid profile and blood pressure of the vitamin D deficiency group were not significantly different from those of the control groups [128]. Various studies have demonstrated that low serum vitamin D levels are correlated to several metabolic conditions, such as elevated TG, low HDL-C, and high blood pressure, in postmenopausal women [129].

Interestingly, vitamin D has been reported to be important not only in alleviating metabolic diseases, but also in improving the quality of life in postmenopausal women [130]. The intake of calcium, vitamin D, and insulin along with isoflavones is reported to have a positive effect on the quality of life parameters (hot flashes, anxiety, and depressive symptoms), sexual life [131], body composition, and metabolic parameters in menopausal women. The ingestion of equal and resveratrol is also recommended to improve the quality of life parameters, such as dryness of vagina, heart discomfort, and sexual problems, in healthy postmenopausal women [132].

However, the role of vitamin D supplementation in preventing the alteration of lipid metabolism in menopausal transition and postmenopausal women needs to be investigated further. 

#### 4.2.2. Dietary Reference Intake (DRI) and Food Sources of Vitamin D

DRI recommendations for vitamin D among adults are as follows: 5 μg/day (19–50 years), 10 μg/day (51–70 years), 15 μg/day (>70 years). The tolerable upper vitamin D intake for adults is 50 μg/day [133]. In the USA and Canada, milk (fluid, dried, or evaporated) is fortified with vitamin D. Furthermore, 3/4 cup of enriched cereal, 3 oz (85 g) of sardines, 3 oz of salmon, 3 oz of tuna (light, canned), 1 tablespoon of cod liver oil, and 1 cup of fortified milk contain 1, 5.8, 15.3, 5.7, 11, and 3.2 μg of vitamin D, respectively. Additionally, exposure to sunlight promotes vitamin D synthesis in the skin [134]. 

#### 4.2.3. Omega-3 Fatty Acids

The human body can use carbohydrate, fat, or protein to synthesize almost all fatty acids, except linoleic acid and linolenic acid [135]. The human cells cannot synthesize these two polyunsaturated fatty acids (PUFAs) from starch. Moreover, the cells cannot convert linoleic acid to linolenic acid and vice versa [135]. Therefore, linoleic and linolenic acids are essential fatty acids that must be obtained from the diet.

Linoleic acid is a polyunsaturated omega-6 fatty acid family member that belongs to one of the two families of essential fatty acids. Thus, the human body cannot synthesize linoleic acid from other nutrients. If linoleic acid is consumed as a part of the diet, the human body can synthesize other necessary omega-6 fatty acids, such as arachidonic acid, prostaglandins, leukotrienes, and thromboxane [136]. Among them, arachidonic acid is the starting material for the synthesis of several eicosanoids. Omega-6 fatty acids are abundant in various nuts and fatty seeds, such as flax seeds, poppy seeds, sesame seeds, and their derived vegetable oils [137].

Linolenic acid is a polyunsaturated omega-3 fatty acid family member. Linolenic acid is an essential fatty acid and thus must be acquired through dietary intake. Both linoleic and linolenic acid fatty acids are members of a family of fatty acids, namely eicosapentaenoic acids (EPAs; 20:5 omega-3 fatty acid) and docosahexaenoic acid (DHA; 22:6 omega-3 fatty acid), that are of great interest to scientists in relation to CVDs and human development. Although the human body can synthesize a very small amount of these omega-3 fatty acids, they can be supplied through the consumption of oily fishes (cod liver, herring, mackerel, salmon, menhaden, and sardine) or fish oils. These essential fatty acids serve as raw materials for the synthesis of eicosanoids that function as hormones. EPA is a PUFA that acts as a precursor for prostaglandin-3 (that inhibits platelet aggregation), thromboxane-3, and leukotriene-5 eicosanoids. Eicosanoids affect multiple human body functions, such as the contraction and expansion of blood vessels or muscles, blood coagulation, regulation of blood lipid metabolism, and immune response to injury and infections, such as fever, inflammation, and pain, by slowing the synthesis [138]. 

Omega-3 fatty acids exert anti-inflammatory, cardioprotective, and insulin-sensitizing effects, whereas omega-6 fatty acids exert proinflammatory effects and increase the risk of cardiometabolic disorders and cancer [139]. A 25 year follow-up study reported that the supplementation of long-chain omega-3 fatty acid and non-fried fish in young adulthood is inversely associated with the incidence of metabolic syndromes later in life [140]. A 16 year follow-up study involving 84,688 female nurses reported that women with a higher intake of omega-3 fatty acids had a lower risk of CHD. Moreover, the inverse correlation of omega-3 fatty acid intake via fish consumption with CHD deaths (multivariable relative risks for fish consumption five times per week, 0.55 (95% CI, 0.33–0.90) for CHD deaths vs. 0.73 (0.51–1.04) was stronger than that for nonfatal myocardial infarction [141]. Western diets are insufficient in omega-3 fatty acids and contain excessive amounts of omega-6 fatty acids. Additionally, the ratio of omega-6/omega-3 fatty acids in the Western diet ranges from 15/1 to 16.7/1. An omega-6/omega-3 fatty acids ratio of 4/1 is associated with a 70% decrease in total mortality. A lower omega-6/omega-3 ratio was associated with decreased risk of breast cancer in women, while the ratio of 2–3/1 suppressed inflammation in patients with rheumatoid arthritis [135]. Therefore, supplementation of low omega-6/omega-3 fatty acids can be useful in the prevention of CVD, cancer, and inflammation [135]. Postmenopausal women are reported to exhibit lower omega-6/omega-3 fatty acid ratio than premenopausal women. Healthy postmenopausal women exhibited higher omega-3 fatty acid levels than women with diabetes or CHD. A significant shift in increased omega-6 and decreased omega-3 was observed in diabetes and CHD [142]. Recently, omega-3 fatty acids were reported to protect against obesity and metabolic syndrome, including the lipid alteration. Tardivo et al. reported that oral supplementation of 900 mg omega-3 per day significantly decreased BMI, waist circumference, blood pressure, serum triglyceride, interleukin (IL)-6, and insulin resistance in a randomized controlled trial involving 87 postmenopausal Brazilian women [143].

#### 4.2.4. Recommendations for Omega-3 Fatty Acid Intake

The consumption of optimal amounts of omega-3 and omega-6 fatty acids is required for obtaining health benefits from essential fatty acids. In the cells, both types compete for the same metabolic enzymes. Thus, excess omega-6 fatty acids prevent omega-3 fatty acids from interacting with the enzymes that convert them to the required compounds [135]. A previous study suggested that rationing between omega-3 and omega-6 fatty acids can promote health, but this is still controversial [144]. The AHA recommends including two fatty fish servings totaling 8 oz (227 g) a week for a heart-healthy diet [145].

#### 4.2.5. Food Sources of Omega-6 and Omega-3 Fatty Acids

The food sources of linoleic acid, an omega-6 fatty acid, are seeds, nuts, vegetable oils (corn, cottonseed, safflower, sesame, soybean, and sunflower), and poultry fat. Linolenic acid, an omega-3 fatty acid, is abundant in oils (canola, flaxseed, soybean, walnut, wheat germ; liquid or soft margarine made from canola or soybean oil), nuts, and seeds (flaxseeds, walnuts, soybeans). The food sources of omega-3 EPA and DHA are human milk, fish, and seafood (>500 mg per 3.5 oz serving), European seabass (bronzini), herring (Atlantic and Pacific), mackerel, oyster (Pacific wild), salmon (wild and farmed), sardines, toothfish (includes Chilean seabass), and trout (wild and farmed). Black bass, catfish (wild and farmed), clam, cod (Atlantic), crab (Alaskan king), croakers, flounder, haddock, hake, halibut, oyster (eastern and farmed), perch, scallop, shrimp (mixed varieties), sole, swordfish, and tilapia (farmed) provide 150–500 mg omega-3 EPA and DHA per 3.5 oz serving. Cod (Pacific), grouper, lobster, mahimahi, monkfish, red snapper, skate, triggerfish, tuna, and wahoo provide less than 150 mg per 3.5 oz serving [135].

#### 4.2.6. Antioxidants and Their Food Sources

Antioxidants are compounds that protect biomolecules from oxidation-mediated damage. Oxidation can potentially damage the chemical components of the normal cell. Oxidative stress is defined as a derangement in the balance between oxidants and antioxidants, which damages biological structures and contributes to disease progression [146]. Oxidative stress is reported to cause damages to the cellular components, such as proteins, lipids, and DNA [147], and increases the levels of proinflammatory cytokines, such as tumor necrosis factor and IL-1*β* [146]. Moreover, oxidative stress is associated with various diseases, such as obesity, diabetes, metabolic syndrome, insulin resistance, atherosclerosis, and CVD, as it is closely associated with chronic inflammation and endocrine function of adipocyte [148]. Most recent studies have reported that estrogens exert antioxidant activity by modulating the expression of antioxidant enzymes [6]. Sack et al. reported that endocrine disruption in systemic oxidative stress at menopause could be attributed to a change in the levels of E2, which exerts antioxidant activity [149]. Postmenopausal women administered with E2 exhibited increased resistance to LDL oxidative modification and pro-atherogenic transformation. However, this antioxidant action was reversed upon discontinuation of E2 administration [149]. Several studies have reported that E2 dose-dependently inhibits the oxidation of LDL in various ex vivo models [150]. Furthermore, E2 is reported to decrease the concentration of atherogenic plasma lipids, ameliorate endothelial function and inflammatory homeostasis, and increases neuronal survival at the metabolic level [151]. Therefore, postmenopausal women usually have less effective antioxidative defense due to the loss of estrogens. Postmenopausal women are recommended to increase their intake of exogenous antioxidants, such as vitamins (vitamin A, *β*-carotene, vitamin C, and vitamin E), plant flavonoids, and soy isoflavones. These nutrients are commonly found in fruits, vegetables, soybean, cocoa, and tea leaf extracts. The highest antioxidant content is found in almonds [152], artichokes, blackberries, blueberries, cherries (sour), chokeberry [153], chocolate (dark, unsweetened), cloves, ground cranberry juice, coffee, cranberries, grape juice, cranberry juice, pomegranate juice, pecans, raspberries, spinach, strawberries, walnuts [154], and red wine [134]. Recently, the administration of olive leaf extract [155] and Hawthorn fruit extract [156] was reported to improve serum lipid profiles in OVX mice via elevating mRNA expression of adiponectin and PPAR-α. Generally, antioxidants have characteristic molecular structures with multiple bonds and aromatic rings. This enables antioxidants to function as reducing agents and to terminate oxidative chain reactions by binding free radicals and inhibiting various oxidative reactions, such as lipid peroxidation [6]. Free radicals are atoms or molecules with one or more unpaired electrons that render the atom or molecule unstable and highly reactive [157]. Lipid peroxidation is initiated on the double bonds of plasma membrane fatty acids via free radical attack. The lipid peroxidation products are highly reactive and dose-dependently affect cell signaling, protein, and DNA [146]. Therefore, the oxidative stress-related diseases in postmenopausal women can be prevented by oral administration of antioxidants. The oral administration of vitamin C (ascorbic acid) and vitamin E (alpha-tocopherol) decreases TC, LDL-C, TG, lipid peroxide, glucose, and glycated hemoglobin levels in diabetic and nondiabetic postmenopausal women receiving hormone therapy [158]. This indicated that the co-administration of both vitamins can further enhance the defense against oxidative stress, not only in the aqueous phase, but also in the lipid phase of the mitochondrial membranes [159]. Supplementation with antioxidative vitamins A, C, E, and omega-3 fatty acids significantly decreased serum lipid peroxide levels in Korean postmenopausal women with hypercholesterolemia. Furthermore, the dietary intervention might be more effective than hormone replacement therapy (HRT). Additionally, the group subjected to a combination of HRT and dietary intervention exhibited reduced levels of thromboxane B2 (TBX2), which is a strong prothrombotic factor [160].

#### 4.2.7. Phytochemicals and Probiotics

Phytochemicals are the nutrient components of plants that contain bioactive components, which can alter the physiology of an organism. They have been used in foods for their sensory properties, such as taste, color, texture, and aroma. However, the ability of phytochemicals to regulate physiology is recently gaining attention. Several phytochemicals function as antioxidants, which protect DNA, lipids, and other cellular components from oxidative stress. Some phytochemicals bind to gene sequences to regulate protein synthesis, mimic hormones, and affect blood chemistry [161]. One of the largest phytochemical groups is the flavonoids. These are detected in several plant foods, such as whole grains, vegetables, fruits, nuts, red wine, spices, and even dark chocolate. 

Probiotics are microorganisms that can be used as dietary supplements. Probiotics aid in digesting food, producing vitamins [162], or preventing intestinal infections. Interestingly, recent studies have demonstrated that multistrain probiotic uptake improved iron uptake [163] and positively modified vascular endothelial function among postmenopausal obese women by decreasing arterial stiffness [164]. Here, we summarize various phytochemicals and probiotics among the natural food sources that can exert antioxidant and anticancer effects, attenuate alterations in lipid profile, and protect DNA [165].

1. Alkylresorcinols (phenolic lipids) in grains may exert a protective effect by decreasing the risks of CVD, diabetes, and several cancers. The food sources of alkylresorcinols are whole grain, wheat, and rye [166].
2. Allicin (organosulfur compound) exerts antimicrobial effects that may reduce ulcers and lower blood cholesterol. The food sources of allicin are chives, leeks, garlic, and onions [167].
3. Carotenoids (including beta-carotene, lycopene, lutein, and hundreds of related compounds) act as antioxidants and may reduce the risks of cancer and other diseases. The food sources of carotenoids are highly pigmented fruits and vegetables (apricot, broccoli, cantaloupe, carrots, pumpkin, spinach, sweet potatoes, and tomatoes) [168].
4. Capsaicin inhibits blood coagulation by decreasing the risk of fatal clots in the heart and arteries. The food sources of capsaicin are peppers [169].
5. Curcumin acts as an antioxidant and anti-inflammatory agent, which may decrease blood coagulation and suppress enzymes that activate carcinogens. The food source of curcumin is turmeric, a yellow-colored spice [170].
6. Flavonoids (including flavones, flavanols, isoflavones, and catechins) exert an antioxidant effect. They scavenge reactive oxygen species and carcinogens and bind to nitrates in the stomach, which prevents the conversion to nitrosamines. Additionally, flavonoids inhibit cell proliferation. The food sources of flavonoids are berries, black tea, celery, citrus fruits, green tea, olives, onions, oregano, purple grapes, purple grape juice, soybean, soy products, vegetables, whole wheat, and wine [165].
7. Genistein and daidzein (isoflavones) are phytoestrogens that are structurally similar to the female sex hormone, estrogen and weakly mimic or modulate estrogen in the human body [171]. They inhibit cell proliferation in the gastrointestinal (GI) tract, which may reduce the risk of breast, colon, ovarian, prostate, and other estrogen-sensitive cancers and osteoporosis [172]. The food sources of genistein and daidzein are soybeans, soy flour, soymilk, tofu, textured vegetable protein, and other legume products.
8. Indoles (organosulfur compound) may trigger the production of enzymes that inhibit carcinogen-mediated DNA damage and estrogen activity. The food sources of indoles are cruciferous vegetables, such as broccoli, Brussels sprouts, cabbage, cauliflower, horseradish, mustard greens, and kale [173].
9. Isothiocyanates (organosulfur compounds that include sulforaphane) function as antioxidants, inhibit enzymes that activate carcinogens [174], and activate enzymes that detoxify carcinogens, and thus reduce the risk of breast cancer and prostate cancer. The food sources of isothiocyanates are cruciferous vegetables, such as broccoli, Brussels sprouts, cabbage, cauliflower, horseradish, mustard greens, and kale.
10. Monoterpenes (including limonene) may trigger enzyme production to detoxify carcinogens and inhibit cancer progression and cell proliferation. The food sources of monoterpenes are citrus fruit peels and oils [175].
11. Phenolic acids may promote enzyme production that increases the water solubility and excretion of carcinogens. The food sources of phenolic acids are coffee beans, fruits (apples, blueberries, cherries, grapes, oranges, pears, and prunes), oats, potatoes, and soybeans [176].
12. Resveratrol functions as an antioxidant and can suppress cancer cell growth. Additionally, resveratrol reduces inflammation, LDL oxidation, and blood clot formation. The food sources of resveratrol are red wine, peanuts, grapes, and raspberries [177].
13. Saponins (glucosides) may interfere with DNA replication and prevent cancer cell [178] proliferation and stimulate immune response [179]. The food sources of saponins are soybean, alfalfa sprouts, other sprouts, green vegetables, potatoes, and tomatoes.
14. Tannins act as antioxidants that may inhibit carcinogen activation and cancer progression [180]. The food sources of tannins are black-eyed peas, grapes, lentils, red and white wine, and tea [134].
15. Probiotics promote the digestion of food, produce vitamins [162], or protect probiotic bacteria from intestinal infections. The food sources for probiotics are yogurt and other fermented foods. The multi-strain probiotics also improve insulin resistance, endothelial dysfunction, and iron uptake [164].

#### 4.2.8. Whole Foods: Best Antioxidant Supplements

Generally, the body absorbs nutrients from a complex mixture in foods [181]. If the nutrients are consumed in a purified and concentrated form, they are likely to interfere with the absorption of other nutrients or with the absorption of other nutrients from foods consumed at the same time. These effects are especially well-documented in case of minerals. For instance, zinc disrupts absorption of copper and calcium, iron disrupts absorption of zinc, and calcium disrupts absorption of magnesium and iron [182]. Among vitamins, vitamin C supplements enhance the absorption of iron, which may result in enhanced iron absorption in susceptible individuals. A high dose of vitamin E interferes with the functions of vitamin K, which delays blood clotting and increases the risk of brain hemorrhage [182] and blocks the entry of useful vitamin E isomers into the bone [183]. The vitamin A precursor, beta-carotene, impairs vitamin E metabolism [182]. These interactions serve as a drawback for the use of purified supplements. Therefore, natural, whole foods are the best source of nutrients. 

## 5. Conclusions

Women experience menopause with aging. Menopause may lead to various changes in lipid metabolism due to reduced estrogen secretion. These changes include enhanced fat mass and decreased FFM, which affects the BMR. Additionally, these changes increase the susceptibility to adiposity and obesity. As menopause is associated with altered lipid levels and increased risk of metabolic disorders, including CVD, postmenopausal women must consume recommended diets and beneficial foods. The strength of this study is that the lipid metabolic disorders associated with menopause were comprehensively summarized right from the basic physiological modifications, including alterations in E2 to dysregulated lipid metabolism along with references. However, the limitation of this review is that physical activity, which is a prominent factor affecting metabolic changes in menopausal women, was not reviewed.

The correlation between the menopausal state and alterations in lipid metabolism should be understood to prevent growing problems of obesity-related disorders, including metabolic syndrome. To protect the cellular components from oxidative stress caused due to estrogen deficiency, further studies are needed to develop energy-balanced dietary interventions, antioxidative foods—and their sources—depending on the menopausal status. Menopause is associated with various alterations in lipid metabolism due to estrogen deficiency. Therefore, supplying beneficial foods such as those described in this review will enable postmenopausal women to counteract these changes. 

## Abbreviations

E2	estrogen
E3	estriol
PPARα	peroxisome proliferator-activated receptor α
PPAR*β*	peroxisome proliferator-activated receptor *β*
PGC1α	peroxisome proliferator-activated receptor gamma coactivator 1 α
PCG1*β*	peroxisome proliferator-activated receptor gamma coactivator 1 *β*
ERR1	estrogen-related receptor 1
BMI	body mass index
SWAN	Study of Women’s Health Across the Nation
SAT	subcutaneous adipose tissue
TG	triacylglycerol
TC	total cholesterol
FFA	free fatty acid
AT-LPL	adipose tissue lipoprotein lipase
FFM	fat-free mass
LBM	lean body mass
DEXA	dual-energy X-ray absorptiometry
BMR	basal metabolic rate
REE	resting energy expenditure
VLDLs	very-low-density lipoproteins
LDLs	low-density lipoproteins
HDLs	high-density lipoproteins
LCAT	lecithin-cholesterol acyltransferase
CETP	cholesterol ester transfer protein
CHD	coronary heart disease
FSH	follicle-stimulating hormone
WHILA	Women’s Health in the Lund Area
OVX	ovariectomized
CEBPb	CCAAT-enhancer-binding proteins
ER α	estrogen receptor α
ER *β*	estrogen receptor *β*
Adipo ER α	adipocyte-specific ER α
AHA	American Heart Association
ACC	American College of Cardiology
RDA	Recommended dietary allowance
CVD	cardiovascular disease
DHA	docosahexaenoic acid
EPA	eicosapentaenoic acid
PUFA	polyunsaturated fatty acid
CHD	coronary heart disease
HRT	hormone replaces therapy
TBX2	thromboxane B2
